# Xylitol Antioxidant Properties: A Potential Effect for Inflammation Reduction in Menopausal Women?—A Pilot Study

**DOI:** 10.3390/cimb47080611

**Published:** 2025-08-02

**Authors:** Ilona Górna, Magdalena Kowalówka, Barbara Więckowska, Michalina Banaszak, Grzegorz Kosewski, Olivia Grządzielska, Juliusz Przysławski, Sławomira Drzymała-Czyż

**Affiliations:** 1 Department of Bromatology, Poznan University of Medical Sciences, 3 Rokietnicka St., 60-806 Poznan, Poland; mkowalowka@ump.edu.pl (M.K.); grzegorzkosewski@ump.edu.pl (G.K.); jprzysla@ump.edu.pl (J.P.); drzymala@ump.edu.pl (S.D.-C.); 2 Department of Computer Science and Statistics, Poznan University of Medical Science, 7 Rokietnicka St., 60-806 Poznan, Poland; basia@ump.edu.pl; 3 Department of Bromatology, Doctoral School, Poznan University of Medical Sciences, 3 Rokietnicka St., 60-812 Poznan, Poland; michalina.banaszak@student.ump.edu.pl

**Keywords:** sugar substitutes, xylitol, oxidative stress, antioxidants, postmenopausal women, DPPH, ABTS+

## Abstract

Introduction: Oxidative stress is a key factor in the pathogenesis of many chronic diseases, especially in postmenopausal women. Xylitol, a sugar alcohol with potential antioxidant properties, may affect oxidative balance when used as a sugar substitute. Aim: This pilot study aimed to assess the effect of replacing sucrose with xylitol on serum antioxidant capacity in postmenopausal women. Methods: This study included 34 women aged 50 to 65 years who successively consumed 5 g/d, 10 g/d, and 15 g/d of xylitol. The dietary intervention lasted a total of 6 weeks, with each phase covering a 2-week period. Diet was assessed twice based on a 7-day dietary interview (Diet 6.0, NIZP–PZH, Warsaw). The material for this study was venous blood. Antioxidant capacity was determined using the DPPH radical scavenging method and the ABTS cation radical scavenging method. Results: In both methods, a significant increase in serum antioxidant potential was observed after replacing sugar with xylitol (*p* < 0.0001). An increase in the ability to neutralize free radicals was observed in almost all women studied. Additional analysis of the effect of selected nutrients on the obtained effects of the nutritional intervention showed that the most significant effect could potentially be exerted by manganese, maltose, sucrose, and mercury, and the strongest positive correlation was exerted by vitamin A, retinol, and vitamin E. Although the values obtained in the constructed models were not statistically significant, the large effect indicates potentially significant relationships that could have a significant impact on serum antioxidant potential in the studied group of women. Conclusions: The results suggest a potential role of xylitol in enhancing antioxidant defense mechanisms in menopausal women. Although the sample size was relatively small, this study was powered at approximately 80% to detect large effects, supporting the reliability of the observed results. Nevertheless, given the pilot nature of this study, further research with larger cohorts is warranted to confirm these preliminary observations and to clarify the clinical significance of xylitol supplementation in populations exposed to oxidative stress.

## 1. Introduction

The sensation of a sweet taste and the concept of a sweetener are undoubtedly significant in the history of human existence. Concepts such as “sweetness” and “sweetening” often evoke direct associations with sucrose, customarily referred to as “sugar”. However, many chemical compounds with similar sweetening properties can be used as a substitute for sucrose. These compounds may also have many other valuable properties, finding wide application in the medical, food, pharmaceutical, and cosmetic industries. One of the most promising sweeteners is xylitol. After hydrogenation of highly purified xylose, crystalline xylitol was obtained in as early as 1942 by Wolfrom and Kohn [1], and after gaining knowledge of xylitol’s involvement in indirect carbohydrate metabolism, Mellinghoff, in 1961, first considered its use as a sugar substitute in the diets of people with diabetes. In the late 1960s, xylitol also attracted the attention of dentists for its ability to reduce plaque formation and enamel demineralization [1,2]. Xylitol is now widely used in food products due to its similar relative sweetness and lower caloric value (2.4 kcal/g) compared with sucrose (4 kcal/g) [3].

Oxidative stress (OS), associated with elevated levels of free radicals, can oxidize biomolecules or structurally modify proteins and genes, leading to signaling cascades that initiate inflammatory diseases [4]. Diseases whose etiology is suggested to be related to oxidative stress include, for example, Alzheimer’s disease, Parkinson’s disease, diabetes, cancer, and cardiovascular disease [5]. An increasing number of studies indicate that xylitol may have antioxidant effects. In animal models, xylitol supplementation has been shown to reduce oxidative stress markers, such as lipid peroxides, while increasing the activity of antioxidant enzymes, including superoxide dismutase, catalase, and glutathione, and reducing malondialdehyde. Reduced oxidative damage in tissues and improved overall antioxidant balance in liver, heart, kidney, and pancreas tissues, as well as in serum, have also been demonstrated. These results indicate that xylitol may modulate the body’s defense mechanisms against oxidative stress. [6,7].

In addition to its antioxidant properties, xylitol exhibits antiviral and immunomodulatory properties [8]. Research indicates that its consumption may alleviate the symptoms of certain infections and modulate the body’s inflammatory response [9].

Given the growing interest in the impact of diet on oxidative stress, it is essential to assess whether replacing sucrose with xylitol may provide health benefits among groups particularly vulnerable to metabolic changes. One such group includes postmenopausal women, who experience a significant increase in oxidative stress due to the decline in estrogen levels. This hormonal change leads to decreased activity of key antioxidant enzymes, such as superoxide dismutase and catalase, and an increased production of reactive oxygen species, contributing to cellular damage. Additionally, postmenopausal women often exhibit increased visceral adiposity, which further exacerbates oxidative stress through the secretion of pro-inflammatory cytokines. As a result, postmenopausal women exhibit elevated levels of lipid and protein oxidation markers and reduced antioxidant capacity, which is associated with an increased risk of developing cardiovascular and metabolic diseases, as well as osteoporosis. [10]. Therefore, studies conducted to find new applications for sweeteners such as xylitol may be helpful in the prevention and combating of diseases associated with increased levels of free radicals.

This study aimed to evaluate a dietary modification, introducing xylitol instead of sucrose into the diet, on the serum antioxidant potential of postmenopausal women. The study was a pilot study.

## 2. Materials and Methods

### 2.1. Materials

**Figure 1:**
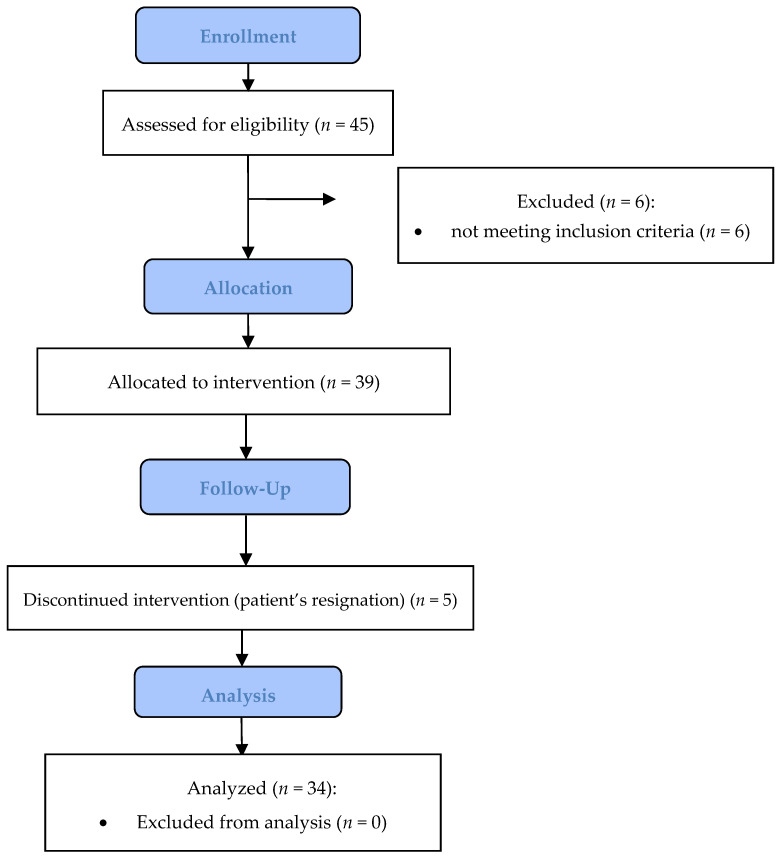
Research block diagram.

This pilot study was conducted in a group of 34 women (Figure 1) meeting the inclusion criteria, including postmenopausal age, use of sugar as a sweetener in coffee or tea at a minimum of 3 teaspoons per day, no use of cholesterol-lowering or glucose-lowering drugs or supplements including those constituting vitamin–mineral complexes, no medical conditions that could affect the results of the study, following a diet other than the traditional nutritional model, and no consumption of xylitol or other sugar substitutes. Medical conditions that excluded the possibility of participating in this study were previous or current cancer (ongoing radiotherapy, chemotherapy); liver, kidney, pancreas, or thyroid disease; acute coronary artery disease; coronary artery transplant or coronary artery bypass surgery; previous myocardial infarction; ischemic or hemorrhagic stroke; congenital metabolic diseases, e.g., phenylketonuria and galactosemia; autoimmune diseases (autoimmune thyroid disease, celiac disease, systemic connective tissue diseases, gastritis, hemolytic anemia, vitiligo, Addison’s disease, hyperbilirubinemia); inflammatory bowel diseases (Crohn’s disease, ulcerative colitis); mental disorders; eating disorders such as anorexia, bulimia, food intolerances, and allergies; and drug and alcohol addiction. This study consisted of three stages, each covering 2 weeks. In the first stage, women who had not previously used xylitol started using it as a replacement sweetener for coffee or tea at 5 g/day while reducing the same amount of used sugar. At each stage of this study (i.e., after every 2 weeks), the xylitol doses increased by 5 g/day to 10 g/day in weeks 3 and 4 and 15 g/day in weeks 5 and 6. Consuming smaller doses of xylitol spread over time, and gradually adapting to its regular consumption significantly reduced the risk of gastrointestinal side effects. Each woman in this study received sachets of weighed xylitol (Danisco Sweeteners, Kotka, Finland) and calendars informing them of their daily xylitol doses and follow-up appointments. The study group of women was asked not to change their lifestyle or diet. All studies were conducted with the approval of the Bioethics Committee of the Poznan University of Medical Sciences—Resolution No. 978/17.

### 2.2. Methods

#### 2.2.1. Nutrition Assessment

The evaluation of the diet was assessed twice: before introducing xylitol into the diet and after the study, to detect any possible significant changes that could influence the results. Food consumption data were collected for 7 consecutive days using a 24 h dietary recall. The food intake was analyzed using Dieta 6.0 software (NIZP–PZH, Warsaw, Poland). The obtained results allowed for the determination of the content of selected nutrients. Nutrient intake was assessed against established nutritional standards for the given population.

#### 2.2.2. Determination of Antioxidant Capacity

The material for this study consisted of venous blood, which was collected from the study women in the morning (at the same time during each collection), fasting (at least 12 h after the last meal), using aspiration/vacuum kits. After transfer to tubes and clot formation, the blood was centrifuged, and the serum obtained was used for assays. The blood was allowed to clot to obtain serum and then centrifuged in a centrifuge at 2500 rpm. The resulting serum was stored in a freezer at −80 °C until assayed. The ABTS (2,2′-azino-bis(3-ethylbenzothiazoline-6-sulfonic acid) cation radical and DPPH (2,2-Di(4-tert-octylphenyl)-1-picrylhydrazyl) free radical scavenging methods were used to determine the antioxidant potential. The percentage of free radical scavenging was calculated from the following formula:

% AA = [(Ak − Ap)/Ak] × 100%

%AA—antioxidant activity; Ak—absorbance of the blank sample; Ap—absorbance of the test sample.

#### 2.2.3. DPPH Assay

Antioxidant capacity was determined using the DPPH assay described by Norma et al. [11]. Briefly, the 0.1 mM methanol solution of DPPH radical (0.8 mL) with 0.01 M phosphate buffer (0.78 mL) and serum (0.02 mL) was incubated in the dark for 30 min. Then, the absorbance was measured at a wavelength of λ = 517 nm (Analytik Jena Spekol 1500, Jena, Germany). The ability of serum to scavenge a free radical was expressed as Trolox equivalent antioxidant capacity (TEAC). The TEAC values were calculated from the Trolox equivalent (TE) calibration curve (y = 3.6343x + 7.7108, R = 0.93). TEAC values were expressed as micromoles of Trolox equivalents per 1 mL of serum (µM TE/1 mL).

#### 2.2.4. ABTS Assay

Antioxidant capacity was determined using the ABTS assay described by Miller et al. [12]. Briefly, the 7 mM methanol solution of ABTS+ radical (2 mL) and serum (0.01 mL) was incubated in the dark for 6 min. Then, the absorbance was measured at a wavelength of λ = 734 nm (Analytik Jena Spekol 1500, Germany). The ability of serum to scavenge a free radical was expressed as TEAC. The TEAC values were calculated from the TE calibration curve (y = 7.5584x + 15.148, R = 0.98). TEAC values were expressed as micromoles of Trolox equivalents per 1 mL of serum (µM TE/1 mL).

### 2.3. Statistical Analysis

#### 2.3.1. Study Design and Sample Size

This pilot study included a sample of 34 postmenopausal women, which was determined based on feasibility and resource constraints rather than formal power calculations. While this sample size limits the generalizability of the results, it provides preliminary data for estimating effect sizes in future larger-scale studies. Post hoc power analysis (conducted using G*Power 3.1) indicated that this study had almost 80% power to detect large effects (Cohen’s d > 0.8) for between-group comparisons at α = 0.05.

#### 2.3.2. Data Description and Assumptions

Data distribution was assessed using the Shapiro–Wilk test for normality, and homogeneity of variances was verified using Fisher’s F-test [13]. Continuous variables are presented as mean ± standard deviation (SD) for normally distributed data or median (Q1; Q3) for non-normally distributed data. Categorical variables are expressed as frequencies (%).

#### 2.3.3. Selection of Statistical Tests

For comparisons between two independent groups:

- Student’s *t*-test was used for normally distributed data.
- Mann–Whitney U test was applied for non-normally distributed data.

For correlation analysis:

- Pearson’s correlation coefficient was used for normally distributed variables.
- Spearman’s rank correlation coefficient was applied for non-normally distributed variables.

#### 2.3.4. Logistic Regression Models

To further evaluate the impact of dietary components on the observed effects, univariate logistic regression models were constructed. Univariate logistic regression was prioritized due to the pilot study’s limited sample size (*n* = 34). The models included nutrients with potential antioxidant or pro-oxidant effects (e.g., vitamins A, E, manganese, mercury). Results are reported as odds ratios (OR) with 95% confidence intervals (CI).

#### 2.3.5. Assessment of Antioxidant Potential: Delta TE Analysis

The antioxidant potential was expressed as the change (delta) in the concentration of µM TE/1 mL before and after using xylitol (the values before and after the dietary modification for ABTS and DPPH methods), where a positive delta value indicates an increase in the ability to scavenge free radicals. The variable “mean delta TE” was analyzed in the following two ways:

(1) As a continuous variable, examining the influence of independent variables on the mean change in TE concentration;
(2) As a binary variable, where, for the calculated delta values, cut-off points, median or mean values were used, depending on the distribution of the analyzed variable.

These cut-off points were the boundary between the obtained effect and its absence, as follows:

- For DPPH assay, delta values ≥ 50 µM TE/1 mL were coded as “effect,” and delta < 50 µM TE/1 mL as “too weak effect or no effect”.
- For ABTS assay, delta values ≥ 15 µM TE/1 mL were coded as “effect,” and delta < 15 µM TE/1 mL as “too weak effect or no effect”.

This approach resulted in subgroups of similar sizes (17 vs. 17 for DPPH and 19 vs. 15 for ABTS) from the initial group of 34 women.

#### 2.3.6. Effect Size Measures

Given the pilot nature of this study, effect sizes (Cohen’s d, correlation coefficients) were emphasized over *p*-values. This makes it easier to plan sample sizes for future experiments.

#### 2.3.7. Software

All analyses were performed using PQStat v1.8.6 (Poznan, Poland), with statistical significance set at *p* < 0.05.

## 3. Results

This study included 34 postmenopausal women aged between 50 and 65 years. Applying the method of spreading smaller doses of xylitol over time and gradually adapting to its regular consumption proved correct. After 6 weeks of use, a survey was conducted during a follow-up visit, which included whether symptoms such as nausea, flatulence, stomach rumbling, watery diarrhea, and increased frequency of bowel movements were noticed while taking xylitol. The results obtained showed that the examined women well tolerated xylitol. Symptoms were reported by 32% of the study participants. The most common was increased frequency of bowel movements (20%), sporadic flatulence (6%), stomach rumbling (3%), and diarrhea (3%). These symptoms most often lasted 1–2 days and appeared after xylitol was included in the diet and each time the dose was changed to a higher one. The results of this study related to the effect of dietary modification by changing the usual sugar used to sweeten warm drinks to xylitol showed, after 6 weeks of use, a significant increase in serum antioxidant potential determined using the DPPH method (Table 1) (*p* < 0.0001) and ABTS+ (Table 2) (*p* < 0.0001). Changes were particularly evident in the DPPH method, where the mean increase was 56.9 ± 37.0 µM TE/1 mL, and the maximum improvement recorded reached 148 µM TE/1 mL (Table 1). In the ABTS+ method, the mean increase was 15.6 ± 20.0 µM TE/1 mL, and the maximum improvement reached 60 µM TE/1 mL (Table 2). Significantly, the increase in free radical neutralizing capacity, as measured by both methods, was observed in almost all the women studied—only one case showed a slight decrease.

Comparison of dietary assessments conducted before and after the intervention revealed no statistically significant changes in the intake of the analyzed nutrients, indicating dietary consistency throughout the study period. Considering the fact that the obtained effect could also be influenced by other factors related to the diet of the studied group of women, it was decided to conduct more detailed analyses. Factors that could influence the obtained effect of increased antioxidant potential of serum after including xylitol in the diet included nutrients that can act beneficially or detrimentally. The beneficial ones included vitamins and minerals with antioxidant character, reducing sugars and additionally sucrose. In turn, the factors that can reduce oxidative potential include heavy metals and cigarette smoking. We used two methods of analysis. The first approach assessed correlations between continuous variables (Table 3 and Table 4). In the second analysis, cut-off points (median or mean value) were used for the calculated delta, which was assumed to be the boundary between the obtained effect and its absence. The obtained groups allowed for the construction of logistic regression models (Table 5 and Table 6).

### Assessment of Effect Size

Based on d-Cohen values, analysis of factors related to the diet of the study group of women that could influence the results obtained for serum antioxidant potential showed that some effects (large and medium) indicate differences between groups. It was noted that the largest effect of influence on the results of the present study, statistically significant, was shown for the manganese content (ABTS: d-Cohen = 0.665, *p* = 0.0461) in daily rations (Table 6). Additionally, high effects were found for maltose (DPPH: d-Cohen = 0.587, *p* = 0.3345) and sucrose (DPPH: d-Cohen = 0.579, *p* = 0.1386), but also for mercury (DPPH: d-Cohen = 0.647, *p* = 0.3015), suggesting that these variables may have a significant effect on serum antioxidant potential in the study group of women. Although the *p*-values were not statistically significant, the large effect indicates potentially significant relationships worth considering. Similarly, for vitamins E (DPPH: Cohen’s d = 0.494, *p* = 0.1620) and A (DPPH: Cohen’s d = 0.488, *p* = 0.4282), the demonstrated medium effect indicates a potential influence that should be confirmed in further, more numerous studies (Table 5).

In turn, taking into account the correlation coefficients, the strongest positive correlation (close to statistical significance) was shown for the content of vitamin A (ABTS: r = 0.338, *p* = 0.0507), which suggests that it could have a significant impact on the obtained results of the antioxidant potential of serum. Relatively high correlations were also found for the content of retinol (ABTS: r = 0.273, *p* = 0.1183) and vitamin E (ABTS: r = 0.278, *p* = 0.1113), which means that antioxidant vitamins play an important role. On the other hand, weak correlations were shown for manganese (ABTS: r = 0.196, *p* = 0.2673) and iron (ABTS: r = 0.208, *p* = 0.2376), while the obtained relationships indicate that it may be worth paying more attention to these components with a larger study group size (Table 4). A detailed analysis of the results of this pilot study showed that, in terms of other nutritional factors, the contents of manganese, vitamin A, and vitamin E in the daily food rations of the studied women could potentially have had the greatest impact on the obtained values of the serum antioxidant potential.

## 4. Discussion

In normal physiology, reactive oxygen species (ROS) and reactive nitrogen species (RNS) production are balanced by enzymatic and non-enzymatic antioxidant mechanisms. If their production exceeds the capabilities of the control mechanisms, OS occurs, which damages cells and tissues. Depending on the duration of oxidative stress and the type of structures exposed to it, it can cause cell ageing due to cell cycle destabilization, cell damage, cell proliferation, or death [14]. Due to the high reactivity of ROS and RNS, they can attack biomolecules that are most important for the organism’s functioning, such as nucleic acids, lipids, and proteins [15]. The irreversible changes induced by radicals in oxidative stress have been identified as a correlative element in the development of many chronic diseases such as neurodegenerative diseases (Huntington’s, Parkinson’s, amyotrophic sclerosis, Alzheimer’s), cardiovascular diseases, cataracts, emphysema, diabetes, and cancers [16]. Research shows that cancer cells are characterized by reduced activity of many antioxidant enzymes, such as superoxide dismutase and catalase, which may damage DNA structure and cancer [17]. Recently, researchers have shown a growing interest in the role of free radicals due to their key importance in various physiological processes and the development of many diseases. In addition to endogenous sources of ROS, there are also factors of exogenous origin, including those related to the profession and the environment, such as chemicals, solar radiation, and ionizing radiation [14]. They also include car exhaust fumes, medications (especially cytostatics), pesticides, excessive alcohol consumption, viruses, bacteria, parasites, and smoking.

It is also worth noting that diet strongly impacts various aspects of human health. One of the more promising substances that can be used as a sugar substitute is xylitol, which has many advantages. Scientific studies indicate that xylitol can exhibit antioxidant properties by scavenging reactive oxygen species. Al-Shahrani et al. [18] showed that agave syrup and xylitol had higher antioxidant properties than all other commercial sweeteners. The antioxidant activity of a number of sugar alcohols against peroxide radicals, hydroxyl radicals, and peroxynitrites was also studied by Kang et al. [19]. They showed that the scavenging capacity of oxygen radicals depends on the number of aliphatic hydroxyl groups in the sugar alcohols of the monosaccharide, which protects cells from oxidative stress by scavenging oxygen radicals. In turn, in an in vitro animal model study, Kang et al. [20] assessed not only the antihyperglycemic effect of various sugar alcohols but also antioxidant activity and oxygen radical absorbance capacity (ORAC). They showed that the highest α-glucosidase inhibitory activity characterized xylitol. The antioxidant properties of xylitol have also been observed previously in studies by, among others, Chukwuma et al. [6] and Naknaen et al. [21]. In a study by Hwang et al. [22], it was also observed that chokeberry jam with added xylitol showed a higher DPPH free radical scavenging capacity (74.31 ± 0.47%) than the same jam with added sucrose (73.71 ± 0.88%) or erythritol (73.48 ± 0.34%). Additionally, in a study conducted by Lee et al. [23], the positive effect of xylitol on the antioxidant potential of ginger tablets prepared with different sweeteners was reported. Tables prepared with xylitol, whose free radical scavenging capacity was determined by the DPPH method, were characterized by an AA% approximately 20% higher than when sucrose was used, and using the ABTS method, the difference was at a similar level [23]. The demonstrated antioxidant properties and, at the same time, the low glycemic response favorable to people with diabetes could also be used to produce food for this group of patients. According to a research by Rutkowska et al. [24], the addition of xylitol instead of sucrose to baking significantly (*p* < 0.05) slowed down the decrease in the concentration of the preservative related to storage time. Additionally, it showed a protective effect on polyphenols, especially anthocyanins, which improved muffins’ antioxidant potential and increased their consumption safety [24]. Recent studies indicate that using xylitol in other food products also brings beneficial effects. Wang et al. [25] developed bifunctional xylitol-based carbon dots (xβ-CD) using 2-hydroxypropyl-β-cyclodextrin (2-HP-β-CD). After their application to rainbow trout fillets, their superantioxidant effect was demonstrated (scavenging of DPPH, ABTS+, and OH radicals), similar to vitamin C, as well as high antibacterial properties, contributing to the extension of shelf life by preventing bacterial infections, protein oxidation, and lipid oxidation.

Based on the performed analyses, it was shown that, in this pilot study, the results obtained did not reach statistical significance due to the limited sample size (*n* = 34). This is common in exploratory studies, where the main objective is not to demonstrate statistical significance but to estimate effect sizes and collect preliminary data for planning future studies with appropriate statistical power. Therefore, in analyzing the results, we focused primarily on evaluating the effect sizes obtained, such as correlation coefficients, d-Cohen measures, and odds ratios (OR) with confidence intervals, which provide valuable information on the direction and strength of the observed relationships. In this study, effect sizes provide a valuable guide for planning future research, helping to determine which variables are worth including in subsequent analyses with a more significant number of study participants. High values of d-Cohen and correlation coefficients suggest that a factor may play an important role and should be further investigated. This allows for better design of future experiments and increased statistical power. In the d-Cohen values and correlation coefficient analysis, special attention should be paid to the potential influence of antioxidant vitamins and minerals on the serum antioxidant potential of the studied group of women after replacing sugar with xylitol. Considering the positive correlation of vitamins A and E and the average effect size, the presence of these vitamins in the diet may be important in reducing oxidative stress in postmenopausal women. As a potent antioxidant, vitamin A can neutralize free radicals, which may improve the results of serum antioxidant activity measurements. Additionally, vitamin E, known for its protective effect on lipids against oxidation, may play a key role in maintaining the stability of cell membranes and preventing oxidative damage in the body [26]. Similarly, for manganese, the large effect size indicates that it may significantly impact antioxidant capacity. Manganese is a key cofactor for manganese superoxide dismutase (MnSOD), an important enzyme in neutralizing free radicals [27]. Iron, on the other hand, although it showed a weak correlation, may also be essential in the context of modulation of antioxidant activity, especially in the case of oxidative disorders associated with abnormal iron metabolism [28]. Additionally, the weaker correlation may be due to the low content of this mineral in the diet of the study group or other variables that affect its metabolism. In conclusion, especially vitamin A and manganese, showing medium and large effect sizes, may play a key role in protection against oxidative stress, and further studies may help better understand their role in the prevention and treatment of oxidative stress-related diseases. The results also showed that the serum antioxidant potential of the women studied may have been influenced by the mercury content of the diet, contributing to a reduction in the effect obtained after changing the sugar to xylitol. It is possible due to the fact that this mineral has the ability to indirectly induce oxidative stress through the formation of complexes, especially with thiol groups and nitrogen groups, which indirectly results in a decrease in the activity of the enzymatic antioxidant system and the formation of ROS [29].

## 5. Limitations

The pilot study carried out was characterized by certain limitations. The amount of xylitol used instead of sugar increased gradually every fortnight. This system of doses was created because, due to only partial absorption, xylitol may have an osmotic laxative effect depending on the dose and individual tolerance related to the presence of specific intestinal microflora [30]. This diversity of the intestinal microflora of the studied women could, therefore, influence xylitol tolerance. Studies indicate that a single intake of 20 g of xylitol may cause nausea, and larger doses (35 g and 50 g) significantly increase the frequency of watery stools, flatulence, and colic [31], which is why the maximum dose of xylitol in these studies was 15 g. Consumption of smaller doses of xylitol spreads over time, and gradual adaptation to its regular consumption significantly reduces the risk of side effects from the gastrointestinal tract [32]. Xylitol is also not recommended for diseases that cause diarrhea, such as irritable bowel syndrome (IBS), Crohn’s disease, or ulcerative colitis, and is one of the products excluded from the FODMAP diet (fermentable oligo-, di-, mono-saccharides, and polyols) [33,34]. Although the gradual adaptation model used in these studies minimized the risk of side effects, future studies could also focus on dose standardization.

Another significant limitation was the lack of a control group, but introducing it at this stage, with limited resources, could significantly increase the costs of the study. Therefore, the authors decided to use the pre–post design and focused on assessing the response to the dietary intervention, treating this study as a starting point for designing subsequent, more advanced, randomized, and placebo-controlled studies

In addition, there are several other limitations. The women studied may have used specific doses of xylitol irregularly and unknowingly inaccurately reported the amount and type of meals consumed in the dietary interviews. In future studies, more advanced monitoring methods, such as mobile applications, should be considered to increase food intake accuracy.

In the present study, low statistical power, resulting from the small sample size, limits the possibility of detecting statistically significant differences in the regression models used, even when observing moderate effects. It is worth emphasizing that the authors assumed that the value considered an “effect” was based on the mean value or median delta. However, despite the lack of statistical significance, the obtained results may be an important guide for designing future studies. For example, the d-Cohen values and confidence intervals for odds ratios suggest that some variables, such as manganese and vitamin A levels, could potentially affect serum antioxidant capacity, which requires further verification in a larger sample. In pilot studies, it is crucial not only to look for statistical significance but also to identify trends and estimate effect sizes, which can help determine the necessary sample size in future experiments.

Scientists have long been looking for an explanation of the properties of xylitol and have found that, during xylitol metabolism, antioxidant capacity is generated by generating NADPH, which results in maintaining the active glutathione antioxidant system, as well as reducing the ratio of oxidized/reduced glutathione [35]. Recent studies using human monocytic cells have further demonstrated that xylitol reduces oxidative stress markers, increases superoxide dismutase activity and glutathione levels, and activates the Nrf2/HO−1 antioxidant pathway, highlighting its potential to modulate cellular redox balance in conditions such as atherosclerosis [36]. In our study, the antioxidant properties of xylitol were assessed using only two colorimetric methods (due to financial constraints also), but they are widely accepted and recognized. However, we would like to point out that the use of DPPH and ABTS methods in studying antioxidant activity in biological matrices is noted in the scientific literature. At the same time, research is still underway to better use and standardize these methods, and the scientific community continues to use DPPH and ABTS assays to obtain meaningful and comparable results. The obtained results, despite their preliminary nature, constitute an important step towards a better understanding of the effect of replacing sugar with xylitol on antioxidant parameters in postmenopausal women and may be the basis for planning more detailed studies covering a larger, more significant number of parameters, including the concentration of antioxidant enzymes, with appropriate statistical power. Therefore, the presented results constitute a chance for effective prevention and perhaps support in treating diseases associated with increased levels of free radicals, as well as encourage further exploration of the antioxidant properties of xylitol. At the same time, it is worth remembering that the obtained preliminary results may not represent the entire population of postmenopausal women.

## 6. Conclusions

A pilot clinical study demonstrated a significant increase in the ability to neutralize free radicals (*p* < 0.0001) in almost all women studied. Analysis of the effect of selected nutrients on the results indicated that, despite the lack of statistical significance in the assumptions made, manganese, maltose, sucrose, and mercury may have had the greatest effect on the results, and vitamin A, retinol, and vitamin E had the strongest positive correlation.

The results indicate that xylitol can be valuable in postmenopausal women’s diet, especially in supporting the body’s fight against oxidative stress. In this population group, a natural decrease in antioxidant capacity is observed, which increases the risk of developing chronic diseases such as atherosclerosis, osteoporosis, or neurodegenerative diseases.

In addition, the use of xylitol as a sucrose substitute may not only reduce sugar intake but also contribute to improving the body’s oxidative–antioxidant balance. Its antioxidant properties suggest that it may support the prevention of lifestyle diseases, including heart disease and type 2 diabetes, especially in populations with increased metabolic risk. Therefore, the study’s results suggest the need to include xylitol as part of the dietary recommendations for postmenopausal women, as it may benefit people with limited glucose tolerance or insulin resistance.

Furthermore, due to the observed health-promoting potential, xylitol could be considered in the future as a functional ingredient in dietary supplements intended for this this age group. However, further studies with a larger group of participants are necessary to confirm the results and explain the mechanisms of xylitol action at the molecular level.

## Figures and Tables

**Table 1:** Serum antioxidant potential of the study group of women using xylitol, determined by the DPPH assay (µM TE/1 mL).

	Before Changing Sugar to Xylitol		After Changing Sugar to Xylitol		Total Change in Value
Analyzed parameter	%AA	µM TE/1 mL	%AA	µM TE/1 mL	Delta (µM TE/1 mL)
X ± SD	20.2 ± 1.43	172 ± 19.6	24.4 ± 0.327	229 ± 4.50	56.9 ± 37.0
Coefficient of variation (%)	7.04	11.4	1.34	1.96	0.65
Median	20.0	168	25.6	246	49.5
Minimum	8.86	15.8	12.3	63.6	−47.6
Maximum	27.0	265	32.9	346	148
*p*-Value	*p* < 0.0001				

%AA—% antioxidant activity; X—mean; SD—standard deviation.

**Table 2:** Serum antioxidant potential of the study group of women using xylitol, determined by the ABTS assay (µM TE/1 mL).

	Before Changing Sugar to Xylitol		After Changing Sugar to Xylitol		Total Change in Value
Analyzed parameter	%AA	µM TE/1 mL	%AA	µM TE/1 mL	Delta (µM TE/1 mL)
X ± SD	87.1 ± 0.306	953 ± 4.05	88.3 ± 0.490	969 ± 6.49	15.6 ± 20.0
Coefficient of variation (%)	0.351	0.425	0.555	0.670	1.28
Median	88.7	974	90.5	997	17.0
Minimum	75.3	797	70.0	726	−71.0
Maximum	93.2	1034	93.5	1038	60.0
*p*-Value	*p* < 0.0001				

%AA—% antioxidant activity; X—mean; SD—standard deviation.

**Table 3:** Evaluation of the effect relationship of independent variables on the mean change in Trolox concentration (µM TE/1 mL) in the DPPH assay.

**Dependent Variable = Delta µM TE/1 mL Mean**			
**Measures**			
**Mean ± SD** **Median [Q1; Q3]**	56.9 ± 37 49.5 [43; 77.25]		
**Independent variables**		Correlation coefficient	*p*-Value
	Zinc (mg)	0.007	0.9707
**Mean ± SD** **Median [Q1; Q3]**	8.73 ± 2.33 8.7 [7.2; 9.7]		
	Iron (mg)	0.032	0.8578
**Mean ± SD** **Median [Q1; Q3]**	11 ± 3.72 9.9 [8.68; 13.1]		
	Copper (mg)	−0.174	0.3256
**Mean ± SD** **Median [Q1; Q3]**	1.17 ± 0.39 1.14 [0.92; 1.34]		
	Manganese (mg)	0.036	0.8415
**Mean ± SD** **Median [Q1; Q3]**	4.51 ± 1.35 4.11 [3.55; 5.25]		
	Selenium (µg)	−0.109	0.5407
**Mean ± SD** **Median [Q1; Q3]**	48.54 ± 16.47 48.08 [37.54; 54.81]		
	Retinol (µg)	−0.015	0.9310
**Mean ± SD** **Median [Q1; Q3]**	651.65 ± 971.47 348.11 [254.16; 459.67]		
	Vitamin A (µg)	−0.177	0.3154
**Mean ± SD** **Median [Q1; Q3]**	1413.44 ± 1017.13 1071.55 [833.78; 1617.87]		
	β-carotene (µg)	−0.191	0.2784
**Mean ± SD** **Median [Q1; Q3]**	9.75 ± 1.67 9.45 [8.55; 10.36]		
	Vitamin C (mg)	−0.199	0.2580
**Mean ± SD** **Median [Q1; Q3]**	102.08 ± 53.53 96.6 [55.73; 142.93]		
	Vitamin E (mg)	−0.150	0.3973
**Mean ± SD** **Median [Q1; Q3]**	7.32 ± 2.68 7.04 [5.37; 8.75]		
	Sucrose (g)	−0.153	0.3863
**Mean ± SD** **Median [Q1; Q3]**	45.71 ± 20.04 40.86 [29.58; 59.75]		
	Glucose (g)	−0.086	0.6288
**Mean ± SD** **Median [Q1; Q3]**	9.68 ± 6.27 6.96 [5.57; 12.31]		
	Galactose (g)	−0.147	0.4078
**Mean ± SD** **Median [Q1; Q3]**	0.64 ± 0.71 0.42 [0.17; 0.93]		
	Lactose (g)	0.074	0.6766
**Mean ± SD** **Median [Q1; Q3]**	8.58 ± 4.78 7.82 [4.63; 11.57]		
	Maltose (g)	−0.156	0.3769
**Mean ± SD** **Median [Q1; Q3]**	1.26 ± 0.33 1.16 [1.08; 1.42]		
	Fructose (g)	0.024	0.8928
**Mean ± SD** **Median [Q1; Q3]**	12 ± 8.36 8.95 [7.31; 13.1]		
	Lead (µg)	−0.050	0.7783
**Mean ± SD** **Median [Q1; Q3]**	54.63 ± 18.17 50.94 [43.29; 60.64]		
	Cadmium (µg)	0.041	0.8201
**Mean ± SD** **Median [Q1; Q3]**	13.09 ± 4.98 12.03 [10.63; 13.14]		
	Mercury (µg)	−0.030	0.8644
**Mean ± SD** **Median [Q1; Q3]**	738.21 ± 995.45 384.27 [347.13; 478.74]		

Q—quartile.

**Table 4:** Evaluation of the effect relationship of independent variables on the mean change in Trolox concentration (µM TE/1 mL) in the ABTS assay.

**Dependent Variable = Delta µM TE/1 mL Mean**			
**Measures**			
**Mean ± SD** **Median [Q1; Q3]**	15.65 ± 20.01 17 [6.5; 25.5]		
**Independent variables**		Correlation coefficient	*p*-Value
	Zinc (mg)	0.155	0.3814
**Mean ± SD** **Median [Q1; Q3]**	8.73 ± 2.33 8.7 [7.2; 9.7]		
	Iron (mg)	0.208	0.2376
**Mean ± SD** **Median [Q1; Q3]**	11 ± 3.72 9.9 [8.68; 13.1]		
	Copper (mg)	0.089	0.6176
**Mean ± SD** **Median [Q1; Q3]**	1.17 ± 0.39 1.14 [0.92; 1.34]		
	Manganese (mg)	0.196	0.2673
**Mean ± SD** **Median [Q1; Q3]**	4.51 ± 1.35 4.11 [3.55; 5.25]		
	Selenium (µg)	−0.175	0.3229
**Mean ± SD** **Median [Q1; Q3]**	48.54 ± 16.47 48.08 [37.54; 54.81]		
	Retinol (µg)	0.273	0.1183
**Mean ± SD** **Median [Q1; Q3]**	651.65 ± 971.47 348.11 [254.16; 459.67]		
	Vitamin A (µg)	0.338	0.0507
**Mean ± SD** **Median [Q1; Q3]**	1413.44 ± 1017.13 1071.55 [833.78; 1617.87]		
	β-carotene (µg)	0.021	0.9042
**Mean ± SD** **Median [Q1; Q3]**	9.75 ± 1.67 9.45 [8.55; 10.36]		
	Vitamin C (mg)	0.147	0.4056
**Mean ± SD** **Median [Q1; Q3]**	102.08 ± 53.53 96.6 [55.73; 142.93]		
	Vitamin E (mg)	0.278	0.1113
**Mean ± SD** **Median [Q1; Q3]**	7.32 ± 2.68 7.04 [5.37; 8.75]		
	Sucrose (g)	0.108	0.5448
**Mean ± SD** **Median [Q1; Q3]**	45.71 ± 20.04 40.86 [29.58; 59.75]		
	Glucose (g)	0.009	0.9610
**Mean ± SD** **Median [Q1; Q3]**	9.68 ± 6.27 6.96 [5.57; 12.31]		
	Galactose (g)	0.201	0.2546
**Mean ± SD** **Median [Q1; Q3]**	0.64 ± 0.71 0.42 [0.17; 0.93]		
	Lactose (g)	0.198	0.2620
**Mean ± SD** **Median [Q1; Q3]**	8.58 ± 4.78 7.82 [4.63; 11.57]		
	Maltose (g)	0.081	0.6476
**Mean ± SD** **Median [Q1; Q3]**	1.26 ± 0.33 1.16 [1.08; 1.42]		
	Fructose (g)	0.038	0.8313
**Mean ± SD** **Median [Q1; Q3]**	12 ± 8.36 8.95 [7.31; 13.1]		
	Lead (µg)	0.001	0.9973
**Mean ± SD** **Median [Q1; Q3]**	54.63 ± 18.17 50.94 [43.29; 60.64]		
	Cadmium (µg)	0.038	0.8296
**Mean ± SD** **Median [Q1; Q3]**	13.09 ± 4.98 12.03 [10.63; 13.14]		
	Mercury (µg)	−0.254	0.1479
**Mean ± SD** **Median [Q1; Q3]**	738.21 ± 995.45 384.27 [347.13; 478.74]		

Q—quartile.

**Table 5:** The influence of additional parameters on the antioxidant potential determined by the DPPH assay in the logistic regression model for two groups of women studied, depending on the assumed effect.

Variable	*N* = 17 (>=50) [Effect] Mean ± SD Median [Q1; Q3]	*N* = 17 (<50) [Too Weak Effect or No Effect] Mean ± SD Median [Q1; Q3]	d-Cohen	*p*-Value	OR [95%CI]	*p*-Value
Zinc (mg)	8.73 ± 1.52 8.68 [7.96; 9.55]	8.73 ± 2.97 8.72 [6.81; 10.47]	0.001	0.9971	1 [0.75–1.34]	0.9970
Iron (mg)	11.31 ± 3.86 10.53 [9.09; 13.01]	10.68 ± 3.67 9.65 [8.54; 13.13]	0.167	0.7048	1 [0.87–1.26]	0.6196
Copper (mg)	1.11 ± 0.27 1.11 [0.91; 1.26]	1.23 ± 0.49 1.21 [0.97; 1.39]	0.291	0.4047	0.4 [0.07–2.87]	0.3967
Manganese (mg)	4.43 ± 1.14 4.45 [3.47; 5.17]	4.59 ± 1.56 4.08 [3.68; 5.46]	0.119	0.8497	0.9 [0.55–1.52]	0.7210
Selenium (µg)	46.23 ± 18.96 43.52 [35.23; 51.36]	50.85 ± 13.73 51.07 [40.19; 56.03]	0.279	0.1792	1 [0.94–1.03]	0.4163
Retinol (µg)	507.26 ± 620.89 349.64 [304.75; 432.17]	796.03 ± 1231.54 323.88 [253.14; 581.11]	0.296	0.9451	1 [0.999–1]	0.4067
Vitamin A (µg)	1169.21 ± 602.56 1000.05 [903.4; 1437.52]	1657.68 ± 1282.16 1414.3 [748.68; 1765.87]	0.488	0.4282	0.999 [0.999–1]	0.1876
β-carotene (µg)	9.59 ± 1.7 9.32 [8.08; 10.34]	9.91 ± 1.68 9.59 [8.79; 10.88]	0.186	0.5815	0.9 [0.59–1.35]	0.5789
Vitamin C (mg)	100.98 ± 57.37 88.52 [54.61; 156.69]	103.19 ± 51.15 97.48 [75.61; 119.48]	0.041	0.9061	1 [0.99–1.01]	0.9025
Vitamin E (mg)	6.66 ± 1.82 6.95 [4.91; 7.96]	7.97 ± 3.26 7.12 [5.73; 10.56]	0.494	0.1620	0.8 [0.62–1.09]	0.1644
Sucrose (g)	40.06 ± 16.15 33.42 [28.74; 52.13]	51.36 ± 22.36 45.81 [33.66; 64.26]	0.579	0.1386	1 [0.93–1.01]	0.1080
Glucose (g)	8.34 ± 4.47 6.26 [5.57; 10.78]	11.02 ± 7.57 7.74 [6.02; 13.42]	0.432	0.3014	0.9 [0.81–1.05]	0.2307
Galactose (g)	0.48 ± 0.37 0.45 [0.25; 0.62]	0.81 ± 0.91 0.36 [0.14; 1.14]	0.480	0.7040	0.5 [0.14–1.47]	0.1878
Lactose (g)	8.69 ± 5.55 9.81 [4.03; 11.88]	8.47 ± 4.04 7.65 [5.66; 9.3]	0.046	0.9451	1 [0.88–1.17]	0.8905
Maltose (g)	1.16 ± 0.16 1.15 [1.07; 1.2]	1.35 ± 0.43 1.19 [1.09; 1.59]	0.587	0.3345	0.1 [0.01–1.83]	0.1205
Fructose (g)	11.02 ± 7.68 8.43 [7.79; 10.82]	12.98 ± 9.11 10.89 [6.91; 14.93]	0.233	0.7565	1 [0.89–1.06]	0.4942
Lead (µg)	52.63 ± 21.34 45.35 [40.33; 55.32]	56.63 ± 14.72 51.8 [50.1; 61.96]	0.219	0.1386	1 [0.95–1.03]	0.5210
Cadmium (µg)	12.84 ± 5.17 12 [10.23; 13]	13.34 ± 4.94 12.16 [10.85; 13.19]	0.098	0.5815	1 [0.85–1.12]	0.7697
Mercury (µg)	427.91 ± 180.4 374.78 [354.28; 445.19]	1048.51 ± 1344.11 415.38 [345.68; 1024.79]	0.647	0.3015	0.999 [0.996–1.001]	0.1634
Smoking				0.6012	3.4 [0.32–36.83]	0.3091
yes	3 (17.65%)	1 (5.88%)				
no	14 (82.35%)	16 (94.12%)				

Q–quartile; OR—odds ratio; CI—confidence interval.

**Table 6:** The influence of additional parameters on the antioxidant potential determined by the ABTS assay in the logistic regression model for two groups of women studied, depending on the assumed effect.

Variable	*N* = 19 (>=15) [Effect] Mean ± SD Median [Q1; Q3]	*N* = 15 (<15) [Too Weak Effect or No Effect] Mean ± SD Median [Q1; Q3]	d-Cohen	*p*-Value	OR [95%CI]	*p*-Value
Zinc (mg)	8.83 ± 1.91 8.6 [7.74; 9.46]	8.61 ± 2.84 9.15 [6.79; 9.65]	0.093	0.7885	1 [0.77–1.4]	0.7809
Iron (mg)	10.94 ± 2.7 10.11 [8.99; 12.48]	11.06 ± 4.83 9.49 [7.6; 13.23]	0.032	0.9319	1 [0.82–1.19]	0.9243
Copper (mg)	1.15 ± 0.36 1.11 [0.97; 1.25]	1.2 ± 0.43 1.21 [0.88; 1.38]	0.117	0.5908	0.7 [0.13–4.27]	0.7279
Manganese (mg)	4.89 ± 1.38 4.63 [3.96; 5.33]	4.03 ± 1.18 3.55 [3.22; 4.76]	0.665	0.0461	1.8 [0.94–3.59]	0.0759
Selenium (µg)	43.72 ± 11.09 43.52 [36.25; 51.22]	54.64 ± 20.24 51.28 [41.86; 58.25]	0.693	0.1270	1 [0.9–1.01]	0.0829
Retinol (µg)	734.33 ± 1136.29 358.74 [307.21; 474.44]	546.91 ± 737.2 273.89 [182.76; 412.45]	0.191	0.1550	1 [0.999–1.001]	0.5802
Vitamin A (µg)	1568.65 ± 1144.61 1414.3 [926.26; 1599.51]	1216.84 ± 824.65 1066.39 [626.18; 1476.54]	0.346	0.2981	1 [1–1.001]	0.3293
β-carotene (µg)	9.73 ± 1.68 9.32 [8.69; 10.34]	9.77 ± 1.72 9.61 [8.5; 10.61]	0.022	0.9493	1 [0.65–1.49]	0.9474
Vitamin C (mg)	98.26 ± 55.43 90.14 [51.42; 127.15]	106.92 ± 52.53 98.69 [71.62; 151.16]	0,160	0.6465	1 [0.98–1.01]	0.6352
Vitamin E (mg)	7.85 ± 2.74 7.39 [5.94; 9.46]	6.64 ± 2.54 5.77 [4.54; 8.18]	0.458	0.1940	1.2 [0.91–1.61]	0.1958
Sucrose (g)	47.39 ± 19.62 41.73 [31.46; 65.4]	43.59 ± 21.05 33.66 [29.18; 56.19]	0.188	0.5554	1 [0.98–1.05]	0.5785
Glucose (g)	8.65 ± 4.03 6.67 [5.79; 11.36]	10.98 ± 8.28 7.25 [5.51; 12.92]	0.373	0.8082	0.9 [0.83–1.06]	0.2948
Galactose (g)	0.68 ± 0.74 0.45 [0.34; 0.89]	0.6 ± 0.69 0.3 [0.04; 0.99]	0.107	0.5427	1.2 [0.43–3.17]	0.7515
Lactose (g)	8.91 ± 5.31 9.3 [4.84; 11.54]	8.16 ± 4.15 7.65 [5.23; 9.99]	0.154	0.6596	1 [0.89–1.2]	0.6487
Maltose (g)	1.22 ± 0.23 1.15 [1.1; 1.24]	1.3 ± 0.44 1.18 [1.04; 1.5]	0.218	0.9447	0.5 [0.06–4.21]	0.5256
Fructose (g)	11.34 ± 6.53 9.55 [7.39; 12.99]	12.85 ± 10.41 8.11 [7.35; 13.06]	0.179	0.8896	1 [0.9–1.06]	0.5983
Lead (µg)	49.96 ± 10.08 50.81 [42.5; 55.99]	60.54 ± 24.09 51.8 [46.92; 66.67]	0.600	0.3145	1 [0.91–1.01]	0.1280
Cadmium (µg)	12.19 ± 2.94 12 [10.61; 13.85]	14.22 ± 6.71 12.06 [10.71; 12.85]	0.410	0.6900	0.9 [0.78–1.07]	0.2599
Mercury (µg)	471.98 ± 443.54 374.78 [352.88; 404.18]	1075.44 ± 1365.57 460.75 [338.87; 977.09]	0.627	0.1270	0.999 [0.998–1]	0.1566
Smoking				1	0.8 [0.09–6.17]	0.8013
yes	2 (10.53%)	2 (13.33%)				
no	17 (89.47%)	13 (86.67%)				

Q—quartile; OR—odds ratio; CI—confidence interval.

## Data Availability

The datasets presented in this article are not readily available because the data are part of an ongoing study. Requests to access the datasets should be directed to the corresponding author.

## Abbreviations

The following abbreviations are used in this manuscript:

DPPH	2,2-di(4-tert-octylphenyl)-1-picrylhydrazyl
ABTS	2,2′-azino-bis(3-ethylbenzothiazoline-6-sulfonic acid
OS	oxidative stress
RSV	respiratory syncytial virus
TEAC	Trolox equivalent antioxidant capacity
TE	Trolox equivalent
%AA	% antioxidant activity
X	mean
SD	standard deviation
OR	odds ratios
Q	quartile
CI	confidence interval
ROS	reactive oxygen species
RNS	reactive nitrogen species
ORAC	oxygen radical absorbance capacity
MnSOD	manganese superoxide dismutase
FODMAP	fermentable oligo-, di-, mono-saccharides, and polyols
